# Multiomic biomarkers after cardiac arrest

**DOI:** 10.1186/s40635-024-00675-y

**Published:** 2024-09-27

**Authors:** Victoria Stopa, Gabriele Lileikyte, Anahita Bakochi, Prasoon Agarwal, Rasmus Beske, Pascal Stammet, Christian Hassager, Filip Årman, Niklas Nielsen, Yvan Devaux

**Affiliations:** 1 Cardiovascular Research Unit, Department of Precision Health, Luxembourg Institute of Health, 1A-B rue Edison, 1445 Strassen, Luxembourg; 2 Department of Clinical Sciences Lund, Anaesthesia and Intensive Care, Lund University, Helsingborg Hospital, Svart-brödragränden 3, 251 87 Helsingborg, Sweden; 3 Swedish National Infrastructure for Biological Mass Spectrometry (BioMS), Lund University, Lund, Sweden; 4 Department of Clinical Sciences Lund, Infection Medicine, Lund University, Lund, Sweden; 5 Science for Life Laboratory, Division of Occupational and Environmental Medicine, Department of Laboratory Medicine, National Bioinformatics Infrastructure Sweden (NBIS), Lund University, 22362 Lund, Sweden; 6 Department of Cardiology, Rigshospitalet, University of Copenhagen, Copenhagen, Denmark; 7 Department of Clinical Medicine, University of Copenhagen, Copenhagen, Denmark; 8 Department of Anesthesia and Intensive Care Medicine, Centre Hospitalier de Luxembourg, Luxembourg, Luxembourg; 9 Department of Life Sciences and Medicine, Faculty of Science, Technology and Medicine, University of Luxembourg, Esch-Sur-Alzette, Luxembourg

**Keywords:** Cardiac arrest, Biomarkers, Clinical outcomes, Prognosis, Multiomics, Artificial intelligence, Machine learning

## Abstract

Cardiac arrest is a sudden cessation of heart function, leading to an abrupt loss of blood flow and oxygen to vital organs. This life-threatening emergency requires immediate medical intervention and can lead to severe neurological injury or death. Methods and biomarkers to predict neurological outcome are available but lack accuracy. Such methods would allow personalizing healthcare and help clinical decisions. Extensive research has been conducted to identify prognostic omic biomarkers of cardiac arrest. With the emergence of technologies allowing to combine different levels of omics data, and with the help of artificial intelligence and machine learning, there is a potential to use multiomic signatures as prognostic biomarkers after cardiac arrest. This review article delves into the current knowledge of cardiac arrest biomarkers across various omic fields and suggests directions for future research aiming to integrate multiple omics data layers to improve outcome prediction and cardiac arrest patient’s care.

## Cardiac arrest, a devastating condition

Cardiac arrest, defined as the cessation of all mechanical activity of the heart, is a common event and devastating for patients and their families. Initial resuscitation efforts can often effectively restore cardiac activity, the substantial subsequent morbidity and mortality in resuscitated patients largely stem from cardiac and cerebral dysfunction induced by prolonged whole-body ischaemia [1]. A substantial amount of these patients will die as a consequence of withdrawal of life-sustaining treatment due to neuroprognostication indicating irreversible severe brain injury [2].

Unconscious cardiac arrest patients in the intensive care unit require neuro-prognostication to predict their outcome. Current guidelines recommend a multimodal approach to prognostication that should be performed at least 72 h after return of spontaneous circulation. This includes repeated clinical examination, electrophysiology, blood biomarkers, and neuroimaging. For blood biomarker prognostication, serial measurements of neuron-specific enolase (NSE) are recommended, with increasing values indicating neuronal cell damage and poor prognosis [2]. The prognostic performance of NSE, however, has been shown to be affected by age, haemolysis, and by the presence of tumors producing the protein [3, 4]. In addition, S100B, neurofilament light chain, glial fibrillary acidic protein, and serum tau have been proposed as potential biomarkers, although they are not currently recommended for neuro-prognostication [2].

Survival and long-term functional neurological status are commonly reported to assess neurological sequelae in patients who initially survive a cardiac arrest. Cerebral Performance Category (CPC) scale is a widely used clinician-rated tool, designed to assess neurological status after brain damage. CPC is divided into five categories of performance, ranging from CPC 1 (no/minimal neurological disability) to CPC 5 (death) [5]. Another commonly used functional outcome measure is the modified Rankin Scale (mRS ranging from mRS 0 (no symptoms) to mRS 6 (death) [6]. Both CPC scale and mRS can be dichotomised into good (CPC 1–2; mRS 0–3) and poor outcome (CPC 3–5; mRS 4–6). The latest statement from the International Liaison Committee of Resuscitation suggests using mRS rather than CPC as mRS captures both physical and cognitive disabilities.[7].

## The unmet need for new prognostic biomarkers after cardiac arrest

Existing markers delineated in the previous chapter often encounter significant limitations including variability in prediction accuracy, suboptimal sensitivity and specificity, high costs, and potential for misclassification [8] highlighting the need to explore novel and complementary biomarkers.

**Fig. 1:**
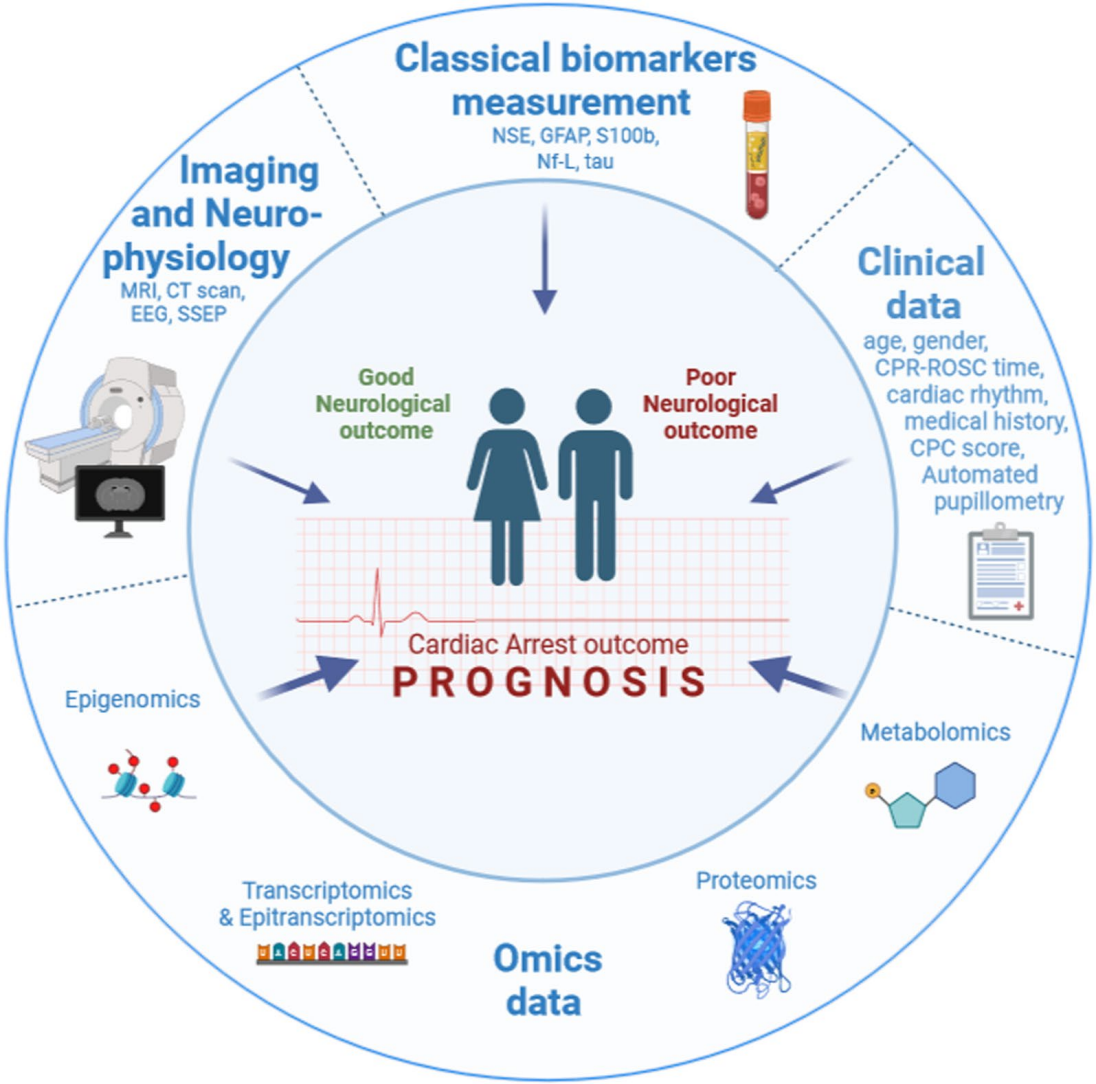
Multicomponent approach for prognostication after cardiac arrest. A multicomponent approach is essential to predict neurological outcome after cardiac arrest (good or poor neurological outcome). Integration of novel omics data with clinical data, classical biomarkers, electrophysiological tests and imaging data, can improve prognostication and guide treatment strategies. Neuron-specific enolase (NSE), calcium binding protein beta (S100b), the glial fibrillary acidic protein (GFAP), neurofilament light (Nf-L), cardiopulmonary resuscitation (CPR), return of spontaneous circulation (ROSC), cerebral performance category score (CPC), magnetic resonance imaging (MRI), computed tomography scanner of the brain (CT scan), electroencephalogram (EEG), somatosensory evoked potential (SSEP)

The Food and Drug Administration defined biomarkers as “measurable indicators of normal biological processes, pathogenic processes, or biological responses to an exposure or intervention, including therapeutic interventions” [9]. High-throughput methods aid in biomarker discovery, identifying gene (DNA and RNA), protein (proteins, peptides, antibodies), metabolism (lipids, carbohydrates, enzymes, metabolites) based-biomarkers [10] to enrich the info provided by histologic, radiographic or physiological markers [9]. To be clinically applicable, a biomarker has to be disease-specific (specificity), easily measurable (sensitivity), indicative of disease progression or treatment response (predictive), rapid, simple, and cost-effective to assess (robust), consistently detectable throughout the day (stable), and measurable non-invasively (e.g., in body fluids like blood, urine, saliva) [11]. Novel biomarkers must provide added value, have undergone thorough validation and standardization processes, exhibit high accuracy, and be cost-effective. A multicomponent approach, integrating omic biomarkers, clinical examination, imaging and electrophysiological techniques, healthcare professionals and researchers may achieve a comprehensive understanding of the molecular and cellular mechanisms responsible for impaired neurological outcome after cardiac arrest. This integration is expected to facilitate early prognostication and personalized patient management (Fig. 1).

## Current and emerging omic biomarkers of cardiac arrest and their assessment

- Epigenomics

The environment and lifestyle can shape epigenetic patterns over time, acting as a kind of cellular memory for past environmental influences. These patterns can vary between cell types and are reversible, potentially influencing disease risk by altering gene activity over the long term [13]. Epigenetic modifications include DNA methylation, histone changes, and noncoding RNAs like microRNAs (miRNAs). MiRNAs regulate key epigenetic factors (e.g., DNA methyltransferases and HDACs) while being controlled by DNA methylation and histone modifications, creating a feedback loop. This interaction, while not strictly epigenetic, modulates gene expression, and its dysregulation can lead to disease. [14].

Mauracher et al*.* studied how neutrophil extracellular traps affect neurological outcomes in out-of-hospital cardiac arrest survivors. They found that higher levels of the neutrophil extracellular traps biomarker citrullinated histone H3, particularly 12 h after admission, correlated with poorer neurological function 30 days later. This suggests that neutrophil extracellular traps, especially citrullinated histone H3, may contribute to brain damage and constitute potential therapeutic targets in cardiac arrest recovery [15]. Histone deacetylase inhibitors can target either multiple classes of HDACs (nonspecific) or individual classes (isoform-specific) by inhibiting the removal of acetyl groups from lysine residues in the NH2 terminal tails of core histones, thus inducing global hyper-acetylation and temporarily altering gene transcription [16]. A study conducted in a rat model of asphyxial cardiac arrest demonstrated that administering intravenous high-dose (300 mg/kg) of valproic acid, a nonspecific HDAC inhibitor, immediately after the return of spontaneous circulation significantly enhances survival rates and neurological outcomes [17].

As far as miRNAs are concerned, a preliminary study provided evidence of elevated levels of circulating miR-21-5p and miR-122-5p in patients experiencing poor neurological outcomes [18]. Subsequently, other investigations, mainly conducted in the TTM-trial [19], reported significant associations between circulating and extracellular vesicle levels of (brain-enriched) miRNAs and neurological outcome and survival after cardiac arrest [20–26].

Analyzing and interpreting epigenomic data are challenging due to cell and tissue specificity, diverse workflows, lack of consensus on analytical methods, and the dynamic nature of the epigenome. [27]. Despite these challenges, studies conducted so far provide convincing arguments that miRNAs and possibly other epigenomic marks may aid in outcome prediction after cardiac arrest [28].

- Transcriptomics

Transcriptomics allows a comprehensive analysis of the structure, composition, functions and regulation of RNA molecules. Diverse RNAs species, including protein-coding RNAs (messenger RNAs) and non-coding RNAs (long non-coding RNAs, circular RNAs, miRNAs…) are expressed in virtually all type of cells and body fluids (blood, plasma, serum, cerebro-spinal fluid, urine, saliva, tear drops), suggesting their value as biomarkers [29]. High throughput technologies such as RNA sequencing and microarrays allow the simultaneous monitoring of thousands of RNA molecules across biological samples. Microarray technology is, however, limited in detection range, cross-hybridization with non-specific targets, and sometimes inaccurate quantification of expression levels [30]. On the other hand, RNA sequencing offers a more thorough and quantitative understanding of the transcriptome, including single-cell profiling [31]. However, sequencing depth, throughput error rates, low abundance transcripts detection and cost are limiting factors [32]. Sequencing also necessitates specific and relatively complex data analysis methods and pipelines. Other targeted methodologies include quantitative PCR, northern blotting, and fluorescence in situ hybridization which complement high throughput transcriptomic methods, aiding in RNA biomarker discovery, validation and functional investigation.

Using RNA sequencing, Stefanizzi et al*.* highlighted an upregulation of the circular RNA circNFAT5 in cardiac arrest patients with poor neurological outcome, suggesting a potential prognostic value when combined with clinical data and established biomarkers of cardiac arrest [33]. Using a murine model of cardiac arrest, Chen et al*,* identified 1162 messenger RNAs and 1920 long non-coding RNAs regulated in the hippocampus, which may be involved in important pathways such as neuronal apoptosis and inflammation [34]. Using machine learning to re-analyze publicly available datasets, Li et al*.* identified RNA interaction networks that could be useful for the identification of potential RNA biomarkers of cardiac arrest [35]. Interestingly, expression levels of several messenger RNAs (including the cold-shock protein RNA-binding motif 3) showed fluctuations upon targeted temperature management in a small group of cardiac arrest patients, suggesting that circulating RNAs may be dynamically regulated. Overall, the prognostic potential of circulating RNAs after cardiac arrest, especially long RNAs, requires further testing in adequately sized patient cohorts. Artificial intelligence and machine learning could aid in this endeavour.

- Epitranscriptomics

Epitranscriptomics, which involves RNA co- and post-transcriptional modifications, impacts gene expression and biological processes (RNA processing, nuclear export, stability, translation…) [36]. Over 170 RNA modifications have been discovered so far and RNA sequencing methods now offer the possibility to study nucleotide modifications [37]. The most frequent and studied RNA modification is named N^6^-methyladenosine *(*m^6^A*)* and emerges as a new player in cardiovascular disease [38]. Adenosine to inosine RNA editing is also currently investigated [39]. So far, RNA modifications have not been addressed in the context of prognostication after cardiac arrest. Table 1 summarizes the pros and cons of common high-throughput methods to study (epi)transcriptomics marks.

- Proteomics

**Table 1:** High-throughput methods to study (epi)transcriptomics marks

Targeted omics	Method	Advantages	Disadvantages
Transcriptomics	Microarray	Simultaneous detection of thousands of genes Cost effective Simple analysis	No detection of novel transcripts Need of transcript specific probes Limited detection of low abundant transcripts Cross hybridization with non-specific targets
	RNA sequencing	Low RNA input Broad dynamic range Low abundant transcript detection + new transcript detection	Long and multiple preparation steps High cost Time consuming Data analysis complexity Error rate
Epitranscriptomics	Direct RNA sequencing	Sequencing of native strands Modifications mapping and quantification Rapid preparation time (2 h) No PCR or fragmentation step Data acquisition in real-time Long reads sequencing High accuracy (96%)	Detects multiple modifications simultaneously Error rate Time consuming, complex data analysis Does not sequence all modifications Chemistry optimization incomplete (no multiplexing) High cost
	Liquid chromatography—mass spectrometry or tandem mass spectrometry	High sensitivity and specificity Quantitative analysis Simultaneous detection of a wide range of components	Global quantification only Transcripts cannot be identified due to enzymatic digestion Modifications should be known upfront Preparation High cost
	Antibody based assays	High sensitivity and specificity Detection of various RNA modifications	Limited modifications (antibodies availability) Modifications should be known upfront Interferences and cross reactivity

As a technique that has been used to identify and quantify molecules in analytical chemistry since more than a century ago, mass spectrometry-based proteomics has gained popularity over the years to discover proteins in a biological context [40]. It has led to the discovery of biomarkers such as neuron specific enolase (NSE) and S100-B, which are associated with poor neurological outcome [41, 42] after CA.

Recently Tandem liquid chromatography mass spectrometry (LC–MS/MS) has emerged as a powerful method to describe the proteomic landscape of various diseases, as it is discovery-based and provides an objective view of the protein composition in the sample. In contrast to other biological sample types, human blood plasma or serum has an extensive quantitative dynamic range where the lowest and highest abundant proteins are estimated to vary between 9 and 13 orders of magnitude [43]. Most common mass spectrometers can confidently detect proteins with 4 orders of magnitude [44], making it challenging to detect low abundant proteins in plasma. While to date discovery-based LC–MS/MS research in the field of cardiac arrest is rather scarce, several studies have aimed to explore the proteomic landscape for the purpose of early prognostication of neurological outcome [45–49]. Table 2 summarizes the features of these studies.

**Table 2:** Proteomic studies in cardiac arrest

Nb. of patients	Sample preparation method	Nb. of proteins	MS method	Regulated proteins	Markers identified	Regulation for poor outcome	Ref
78	Top14 depletion (Multiple Affinity Removal LC Column, Agilent)	885	iTRAQ	55	SERPINA1, AGT	Upregulated at 72 h	[45]
41	Top14 depletion (Multiple Affinity Removal System)	NA	DDA	7	KAIN, AL1A1	KAIN: Upregulated at 24 h and 72 h. AL1A1: Downregulated at 24 h, upregulated at 72 h	[46]
96	Plasma: Top 12 depletion (Abundant Protein Spin Column, Thermo) Brain: Sonication, used protein rich supernatant	Plasma: 299, Brain: 4595	DDA	60	ENO1, YWHAZ, CFL1, HSPA8	NA; The proteins in "Markers identified" combined with clinical parameters applied in multi modal regression model improved prediction of PNO	[47]
11	2D-GE, Top12 depletion + 2D-GE (Pooled samples in good vs poor due to cardiac arrestrce depleted protein material)	MALDI-TOF:11, Triple-Q-LI: 17	Undepleted: MALDI-TOF, Depleted: Triple-Q-LI trap MS	NA (No differential expression analysis done)	Depleted: YWHAZ, Arf-GAP, MKLN1, Undepleted: H1a-A2, HP, Amyloid related serum protein	Proteins identified in Poor outcome found in 2D-GE	[48]
78	Standard neat serum preparation	403	DIA	29 + 6 + 8 = 43	IGFBP2, C7, IGFBP4, ITIH1,PROZ, AFM	Up in all time points: IGFBP2, C7, Up in 24 and 72: IGFBP4, Up in 24: RNASE1, Up in 72: IGHV3-23, Down in 24 and 48: ITIH1, Down in 24 and 72: PROZ, AFM	[49]

2D-GE; Two Dimesional Gel Electrophoresis, iTRAQ; Isobaric tag for relative and absolute quantification, DDA; Data dependent acquisition, DIA; Data-independent acquisition, MALDI-TOF; Matrix-assisted laser desorption/ionization—Time of Flight, Triple-Q-LI; Triple quadrupole—linear ion trap, PNO; Poor neurological outcome, SERPINA1; Alpha-1-antitrypsin, AGT; Angiotensinogen, KAIN; Kallistatin, AL1A1; Retinal dehydrogenase 1, ENO1; α-enolase, YWHAZ; 14-3-3 protein zeta/delta, CFL1; cofilin-1, HSPA8; heat shock cognate 71 kDa protein, Arf-GAP; GTPase ANK repeat and PH domain-containing protein 2, HP; Haptoglobin, MKLN1; Muskelin, H1a-A2; Chain M of the human histocompatibility complex, IGFBP2; Insluin-like growth factor-binding protein 2, C7; IGFBP4; Insulin-like growth factor-binding protein 4, Complement component 7, ITIH4; Inter-alpha-trypsin inhibitor heavy chain H1, PROZ; Vitamin K-dependent protein Z, AFM; afamin. In these studies, patients with good and poor neurological outcome (cerebral performance category score 1–2 vs. 3–5, respectively) have been compared at 24–72 h after cardiac arrest, and markers associated with outcome have been described. Out of the 16 proteins reported to be statistically significantly associated with neurological outcome, only one protein marker, 14-3-3 protein zeta/delta (YWHAZ) was found in two of the studies [47, 48]. This protein has been shown to be elevated in acute myocardial infarction [50]. YWHAZ is believed to possess neuroprotective properties [51], but its role in predicting neurological function is not yet fully characterized. The inconsistency in overlap between the reported markers could be affected by the different mass spectrometry techniques used. For instance, Distelmaier et al*.* [47] used label free quantification coupled with multiple reaction monitoring assay, whereas Boyd et al*.* [48] relied on 2D gel electrophoresis followed by MALDI-TOF

Moreover, the relatively small cohort sizes in the studies seen in Table 2 could also limit robust protein identification. Facilitating high throughput while improving quantitative depth in mass spectrometry proteomics can enable robust discovery of new biomarkers in cardiac arrest at larger scale. Additionally, combining large cohort studies with emerging machine learning methods can accelerate clinical biomarker discovery and improve biological understanding in cardiac arrest.

- Metabolomics

Cardiac arrest is the most extreme case of acute metabolic disturbance. With the abrupt halt of oxygen delivery, the production and levels of adenosine triphosphate decline rapidly. This impairs cell membrane regulation, leading to the generation of reactive oxygen species, cell swelling, apoptosis, and necrosis. Although reperfusion sparks the generation of reactive oxygen species.

Metabolomics is the comprehensive measurement of low molecular-weight molecules (metabolites) in biological fluids.

According to the latest 5.0 update of the Human Metabolome Database [52], 217,290 metabolites have been identified, of which 3408 have been detected in blood. The various chemical characteristics and broad concentration range of metabolites imply that no single technique or platform can detect and quantify all metabolites effectively. Researchers use two main metabolomics approaches: untargeted and targeted. Untargeted metabolomics profiles a wide range of metabolites without prior knowledge, often for biomarker discovery, but faces challenges like unknown metabolite identities and potential false results. Targeted metabolomics quantifies specific metabolites, offering precise pathway analysis but may miss new biomarker discoveries.

Table 3 overall, the field of metabolomics in the context of cardiac arrest is in the early stages, a key observation has been the early elevation of plasma metabolite levels originating from the tricarboxylic acid cycle in patients resuscitated from out-of-hospital or in-hospital cardiac arrest compared to controls [53, 54]. Given the tricarboxylic acid cycle's central role in energy generation under aerobic conditions, these alterations likely mirror the ischemic insult during cardiac arrest. Furthermore, the significance of mitochondria was underscored by the higher plasma levels of acylcarnitines, suggestive of mitochondrial injury due to impaired beta-oxidation of free fatty acids. Conflicting results regarding several amino acids have been reported. In the letter to the editor, Tsai et al*,* found higher levels of alanine to be associated with a higher chance of survival [55]. This was in direct contrast to later studies in which higher levels of amino acids, including alanine, were observed in in-hospital cardiac arrest patients compared to controls and significantly higher in non-survivors [53, 54, 56]. In a recent study, a subset of 60 metabolites characterized distinct early metabolic phenotypes strongly linked with mortality, complementing established early predictors [57]. Few studies report the effects of patient treatment on metabolic profiles. Targeted hypothermia to 33°C, compared with 36 °C, lowered the levels of branched-chain amino acids such as valine and leucine following rewarming [57], while inhalation of xenon did not affect circulating metabolites [56].

**Table 3:** Metabolomics research in cardiac arrest

Type of arrest	Platform	Nb of metabolites evaluated	Nb of patients	Major findings	Ref.
OHCA	MS (untargeted)	137	144 (17 with good neurological outcome)	Higher levels of branched-chain amino acids associated with lower chance of survival. Higher alanine metabolites associated with higher chance of survival	[55]
IHCA	MS (untargeted)	93	13 (all died within 3 days)	Interspecies similarities with cardiac arrest rat model and IHCA patients. Tricarboxylic acid metabolites increased compared to non-cardiac arrest human controls	[54]
OHCA	MS (targeted)	60	163 (54 died within 180 days)	Tricarboxylic acid metabolites higher in non-survivors. Metabolic phenotypes independently associated with higher chance of survival	[53]
OHCA	MS (targeted)	60	146 (43 died within 180 days)	Targeted temperature management influences metabolism for hours after patients’ return to normal temperature. A 33 °C temperature increased tricarboxylic acid cycle metabolite levels and reduced branched-chain amino acids levels compared to 36 °C. Higher levels of branched-chain amino acids were associated with a higher chance of survival	[57]
OHCA	NMR (targeted)	146	105	Xenon does not alter metabolic profile. Higher levels of branched-chain amino acids observed in survivors 24 h from cardiac arrest	[56]

OHCA: out-of-hospital cardiac arrest, IHCA: in-hospital cardiac arrest, MS: mass-spectrometry, NMR: nuclear magnetic resonance spectroscopy

- Imaging and neurophysiology

Besides biomarkers and clinical examination, imaging studies and neurophysiology are part of a multimodal neuroprognostication strategy in comatose cardiac arrest patients [58]. Pros and cons of currently available imaging and neurophysiological tests are presented in Table 4.

**Table 4:** Pros and cons of currently available imaging and neurophysiological tests

Test	Advantages	Disadvantages
Brain computed tomography scans	- Easily performed - Availability in every hospital - Not influenced by sedation	- Needs the transportation of patient - Lack of specificity in moderate cases of hypoxic-ischaemic brain injury - Interpretation by radiologist
Brain magnetic resonance imaging	- High specificity for detecting hypoxic-ischaemic brain injury Not influenced by sedation	- Needs the transportation of patient - Cumbersome in ventilated patients (magnetic field) - Radiology expertise - Not available in every hospital - Expensive and time consuming
Automated pupillometry	- Performed at the bedside - Objective assessment of pupillary reactivity Calculation of a reactivity index - Interpretation of the results by the treating physician - Repeated measurements easily possible	- Uncertain influence of sedation - Calculated indexes need validation in the field of cardiac arrest
Electroencephalography	- Performed at the bedside - Allows for adaptation of the treatment (e.g., epileptic activity) - Continuous measurement possible	- Influenced by sedation - Needs trained personnel for electrode application - Neurologists/neurophysiologists needed for interpretation - Possible high interrater variability in interpretation - Lack of universally used definitions for malignant patterns - Prone to (muscular) artefacts (shivering, myoclonus) - Only possible during “office hours” (for routine electroencephalography in most centers)
Somatosensory evoked potentials	- Performed at the bedside - Probably not influenced by sedation	- Not available at every hospital - Needs trained personnel for performing the test and neurologists/neurophysiologists for interpretation - Only possible during “office hours” in most centers

Native brain computed tomography scans are performed either on admission or after 24–48 h after cardiac arrest and allow cortico-subcortical gray/white matter differentiation ratio analysis. A reduced gray/white matter differentiation ratio is highly specific (close to 100%) and has a sensitivity lower than 50% [59].

Magnetic resonance imaging because of being more challenging in intubated and ventilated patients is often done after 48 h in patients not regaining consciousness. Magnetic resonance imaging mainly focuses on diffusion weight imaging and fluid-attenuated inversion recovery signals (specificity up to 95% and sensitivity of 70%) [59]. More advanced techniques using diffusion tensor imaging have shown promising results (specificity 100%, sensitivity 89%) [60]. Also, gray matter morphometry measuring cortical thickness allows for prognostication (specificity 100%, sensitivity 91%) [61]. The latter techniques are limited by the complexity of data analysis, dependent on specialized research teams and further validation.

Electrophysiology studies encompass electroencephalography and somatosensory evoked potentials. Electroencephalography tracings are classified to continuous background, malignant or even highly malignant patterns and presence or absence of reactivity to stimuli. Using such classifications results in high interrater agreement and a specificity ranging from 90.6 to 100% [62]. More recently, a prospective study on those highly malignant electroencephalography patterns reached a specificity of 97% [63]. In somatosensory evoked potentials, the absence or low amplitude of N20 peak waves indicates a poor neurological outcome with a specificity of 99% (sensitivity up to 49%) [64].

Automated pupillometry is a bedside tool to assess the pupillary light reflex generating indexes used for prognostication at an early stage of admission to the intensive care unit (specificity up to 100%, sensitivity 30%) [65, 66].

Recent efforts aimed to identify combinations of different prognosticators to identify patients who will eventually have a good outcome [67].

With the ongoing research on biomarkers of neurological damage, it might become possible to identify the substrate for the electrophysiological and imaging findings, which might then further strengthen the validity of these tests and elucidate the underlying pathophysiological processes [68].

Although most of the abovementioned tests are important outcome predictors, they must never be used as sole tests. Their interpretation needs to be done in the general clinical context, excluding all confounders and considering the clinical examination and other (multiomic) biomarkers.

## Multiomic analysis, added value of machine learning and artificial intelligence

The advent and extensive adoption of omics technologies, including genomics, transcriptomics, proteomics, metabolomics, and various other omics fields covered in this manuscript, along with advancements in computational capabilities, have opened new avenues for exploring the molecular mechanisms underlying cardiac arrest. The one-size-fits-all approach fails to consider individual factors such as lifestyle habits, environment, genetics, and others, thereby reducing the efficacy of interventions [69]. These factors underscore the need for employing multiomics or big data approaches within this domain, considering recommendations for data integration [70].

Recent research trends indicate a shift from a reductionist to a global approach in the application of multiomic approaches, aiming to leverage the extensive volumes of "single-omics" datasets already widely available [69, 71]. The emergence of data science and advancements in bioinformatic tools have facilitated the integration of data across multiple omic domains. Cardiovascular disease including cardiac arrest presents an opportunity and a need for integrated analysis of multiomics data to identify risk prediction models [72]. Artificial intelligence and machine learning have the capacity to aid not only during the feature selection process but also to reach optimal prediction due to a continuous learning process [73]. This approach enables comprehensive phenotyping and clinically actionable discoveries [74].

## Challenges and future recommendations

Clinically, a combined multimodal approach offers the potential for more personalized and non-invasive medicine, making testing easier and more comfortable for patients (blood, urine, saliva based-biomarkers). The feasibility has also improved with the reduction in device costs and increased portability, simplifying measurements for clinicians and allowing for measurements at the bedside. Additionally, the increase in the speed of the measurement of several omics parameters reduces the need for lengthy analyses, while imaging tests are already widely available. A multicomponent approach—clinical, imaging and multiomics—could soon be implemented in clinical practice to support decision-making, personalize healthcare delivery, and ultimately improve patient outcomes while providing timely information to families.However, the use of multiomics data encompasses several challenges associated with data heterogeneity and integration, one of which is the high dimensionality of the data. Therefore, robust and standardized integrative approaches are needed to address this challenge.

The integration of processed multiomics data into medical records challenges precision medicine. Innovative solutions are required for high data volume, heterogeneous datasets and limited resources. Accurate and cost-effective diagnostic and prognostic approaches and actionable interpretations are needed to streamline patient treatment. Developing, testing and validating a Findable, Accessible, Intelligent, and Reproducible (FAIR) approach will facilitate the successful implementation of precision medicine [75]. Standardized data integration processes are needed to ensure reproducibility of research outputs [70].

Predicting outcomes and treatment effects after cardiac arrest remain unmet clinical needs. Current prognosis markers and methods have limitations. A multicomponent approach, combining multiple omics data layers, imaging, neurophysiology, clinical assessment and machine learning may improve prediction, (Fig. 1). Integrating novel prognostic biomarkers into clinical workflows will.

Despite numerous hopes, the journey from biomarker discovery to clinical applicability is a complex, expensive and time-consuming process. It involves various phases of translational research such as discovery, validation, preclinical research, clinical trials, regulatory approvals, reviews, and safety monitoring. This requires significant resources, expertise, and investment by researchers, clinicians and industries. A partnership between different fields of expertise and public–private partnerships is needed to address the challenge of developing accurate methods to predict outcomes after cardiac arrest. Incentives and investments from research funders are necessary to cross international boundaries to ensure worldwide application of translational research [76].

## Data Availability

No applicable.
